# Marine medaka heat shock protein 90ab1 is a receptor for red-spotted grouper nervous necrosis virus and promotes virus internalization through clathrin-mediated endocytosis

**DOI:** 10.1371/journal.ppat.1008668

**Published:** 2020-07-08

**Authors:** Wanwan Zhang, Kuntong Jia, Peng Jia, Yangxi Xiang, Xiaobing Lu, Wei Liu, Meisheng Yi

**Affiliations:** 1 School of Marine Sciences, Sun Yat-sen University, Guangzhou, Guangdong, China; 2 Southern Marine Science and Engineering Guangdong Laboratory (Zhuhai), Zhuhai, Guangdong, China; 3 Guangdong Provincial Key Laboratory of Marine Resources and Coastal Engineering, Guangdong, China; Washington University in Saint Louis School of Medicine, UNITED STATES

## Abstract

Nervous necrosis virus (NNV) can infect many species of fish and causes serious acute or persistent infection. However, its pathogenic mechanism is still far from clear. Specific cellular surface receptors are crucial determinants of the species tropism of a virus and its pathogenesis. Here, the heat shock protein 90ab1 of marine model fish species marine medaka (MmHSP90ab1) was identified as a novel receptor of red-spotted grouper NNV (RGNNV). MmHSP90ab1 interacted directly with RGNNV capsid protein (CP). Specifically, MmHSP90ab1 bound to the linker region (LR) of CP through its NM domain. Inhibition of MmHSP90ab1 by HSP90-specific inhibitors or MmHSP90ab1 siRNA caused significant inhibition of viral binding and entry, whereas its overexpression led to the opposite effect. The binding of RGNNV to cultured marine medaka hMMES1 cells was inhibited by blocking cell surface-localized MmHSP90ab1 with anti-HSP90β antibodies or pretreating virus with recombinant MmHSP90ab1 or MmHSP90ab1-NM protein, indicating MmHSP90ab1 was an attachment receptor for RGNNV. Furthermore, we found that MmHSP90ab1 formed a complex with CP and marine medaka heat shock cognate 70, a known NNV receptor. Exogenous expression of MmHSP90ab1 independently facilitated the internalization of RGNNV into RGNNV impenetrable cells (HEK293T), which was blocked by chlorpromazine, an inhibitor of clathrin-dependent endocytosis. Further study revealed that MmHSP90ab1 interacted with the marine medaka clathrin heavy chain. Collectively, these data suggest that MmHSP90ab1 is a functional part of the RGNNV receptor complex and involved in the internalization of RGNNV via the clathrin endocytosis pathway.

## Author summary

Nervous necrosis virus (NNV), a *Betanodavirus* belonging to the *Nodaviridae* family, causes severe financial losses to aquaculture industry worldwide, nevertheless, its pathogenic mechanism remains unknown. Virus receptors are pivotal for viruses to cause infection and pathogenicity. However, knowledge regarding the cellular receptor for NNV is limited, and it has been unclear how the receptor participates in intracellular trafficking of NNV particles. In this study, we demonstrated that marine medaka heat shock protein 90ab1(MmHSP90ab1) was a functional receptor of RGNNV for the first time. Furthermore, MmHSP90ab1 expression could independently lead to RGNNV internalization through the clathrin endocytosis pathway. Our findings will contribute to developing novel strategies against NNV infection by targeting the MmHSP90ab1-clathrin endocytosis pathway.

## Introduction

Nervous necrosis virus (NNV), a *Betanodavirus* belonging to the *Nodaviridae* family, is a non-enveloped positive-sense single-stranded RNA virus, which infects various farmed and wild fish species and causes severe financial losses to aquaculture industry worldwide [1, 2]. NNV particles are formed by the only external viral structural protein and two genetic RNA segments [3]. Based on the capsid protein (CP) sequences, NNVs are classified into four genotypes, including Striped jack nervous necrosis virus, Tiger puffer nervous necrosis virus, Barfin flounder nervous necrosis virus and Red-spotted grouper nervous necrosis virus (RGNNV) [4, 5]. Among them, RGNNV was reported as the most commonly detected and widest geographic distributed cluster of NNV [6].

Viruses utilize viral surface protein to bind to specific receptor(s) present on the host cell surface to invade cells and trigger the viral infection and pathogenesis [7]. The specificity of virus-receptor interaction determines the host range, tissue tropism, and viral pathogenesis. Increasing evidence has shown that viral receptors perform different functions on multiple stages of virus life cycle, such as attachment, penetration, transcription, assembly, and release [7–9]. Thus, identification of virus receptors and revealing the mechanism of virus-receptor interaction would be critical for better understanding and controlling viral diseases. To date, a plethora of virus receptors have been identified in diverse cells derived from mammal and reptile [10], however, viral receptors for viruses of aquatic animals were rarely reported. Previously, it was reported that NNV entered SSN-1 cells through receptor-mediated, cell surface sialic acid, micro- and macro-pinocytosis pathways [11], and grouper heat shock cognate protein 70 (HSC70) was identified as a potential receptor or co-receptor for NNV [12]. However, it is hard for only one receptor to complete the whole process of viral entry. Therefore, further studies are needed to identify the specific receptor(s) required for NNV entry.

Heat shock protein 90 (HSP90), a highly conserved molecular chaperone, is a key player in a variety of cellular processes, such as cell cycle control, cell survival, cytoskeletal integrity hormone, cell signaling pathways, and immunological functions [13–15]. The HSP90 family has four isoforms, HSP90α, HSP90β, tumor necrosis factor receptor-associated protein 1 (TRAP1), and glucose-regulated protein 94 (GRP94) [16–18]. HSP90α, HSP90β, and GRP94 have been reported to participate in viral infection and are crucial for several DNA and RNA viruses for viral protein folding, entry, replication, transport, and assembly [19]. HSP90β, present on the surface of Vero cells, is a binding receptor for Japanese encephalitis virus [20]. Human HSP90β facilitates enterovirus 71 viral particles assembly [21]. HSP90α promotes the stability of herpes simplex virus-1 VP16 [22]. As a putative receptor, chicken HSP90α has been proved essential for infectious bursal disease virus (IBDV) entry into DF-1 cells [23]. GRP94 can block hepatitis C virus l-induced apoptosis [24].

So far, several novel and traditional techniques have been applied to identify virus receptors, such as immunoprecipitation (IP) based on mass spectrometry, affinity chromatography, virus overlay protein binding assay, and haploid genetic screen [25–27]. CP, as the only structural protein exposed on the surface of NNV particles, is responsible for the attachment of NNV to its susceptible cells [28]. However, the receptor(s) interacting with CP remains enigmatic. In this study, using the embryonic cell line (hMMES1) derived from the marine model fish marine medaka (*Oryzias melastigma*), we identified a CP interacting protein marine medaka heat shock protein 90 kDa alpha, class B, member 1 (MmHSP90ab1), and mapped the interaction region(s) between CP and MmHSP90ab1. Furthermore, we examined the potential of MmHSP90ab1 acting as a functional co-receptor to facilitate RGNNV entry via clathrin-mediated endocytosis. Collectively, our study will provide a prerequisite for future investigations into precise molecular events involved in NNV entry into permissive cells.

## Results

### MmHSP90ab1 interacts with CP

**Fig 1:**
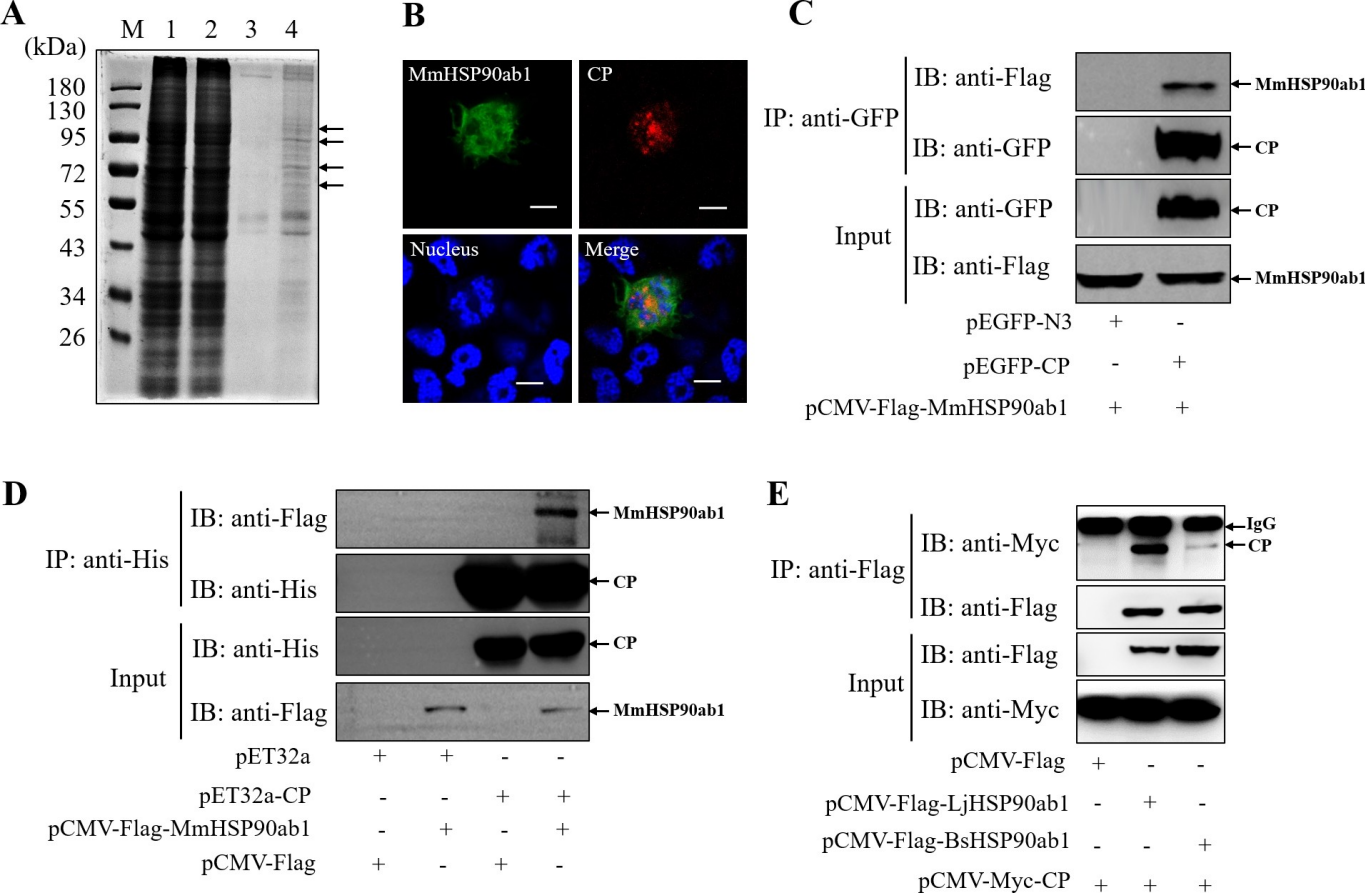
Marine medaka HSP90ab1 (MmHSP90ab1) interacts with RGNNV capsid protein (CP). (A) The lysates of hMMES1 cells transfected with *pEGFP-N3* (Lane 1) or *pEGFP-CP* (Lane 2) plasmids. The precipitated cell proteins with affinity to GFP (lane 3) or GFP-CP (lane 4) were separated by 10% SDS-PAGE and stained with Coomassie blue. Lane M, protein marker. Black arrows indicated the specific bands for mass spectrometry. (B) HEK293T cells were cotransfected with *pCMV-Flag-MmHSP90ab1* and *pCMV-Myc-CP* plasmids. MmHSP90ab1 (Green) and CP (Red) were detected by immunofluorescence staining with anti-Flag or anti-Myc abs, respectively. Nucleus were stained by Hoechst 33342, Bar = 10 μm. (C) Lysates from HEK293T cells transfected with *pCMV-Flag-MmHSP90ab1* and *pEGFP-C*P or *pEGFP-N3* were subjected to immunoprecipitation with anti-GFP abs. Immunoprecipitates and whole-cell lysate (Input) were immunoblotted with anti-GFP and anti-Flag abs. (D) The lysates of HEK293T cells transfected with indicated plasmids were pulled down with purified His-CP or His proteins. The proteins bound to CP and the input were immunoblotted with anti-His and anti-Flag abs. (E) *pCMV-Flag-LjHSP90ab1* or *pCMV-Flag-BsHSP90ab1* and *pCMV-Myc-CP* were cotransfected into HEK293T cells. The cell lysates were immunoprecipitated with anti-Flag abs. The immunoprecipitates and input were immunoblotted with indicated antibodies.

To identify CP-interacting proteins, we performed an IP assay using anti-GFP antibodies (abs) in *pEGFP-N3* and *pEGFP-CP* plasmids transfected hMMES1 cells, respectively. Coomassie blue staining for SDS-PAGE of the immunoprecipitation showed that lots of specific bands were observed in precipitated proteins of *pEGFP-CP* transfected cells compared to the control group (Fig 1A). Then, gels of these bands were analyzed by mass spectrometry, and several candidates of CP-interacting proteins were identified (S1 Table). Among them, MmHSP90ab1 was selected for following studies due to its important role during virus infection. First, Co-IP assay was carried out to confirm the interaction of MmHSP90ab1 and CP. As shown in Fig 1B and 1C, MmHSP90ab1 colocalized and coprecipitated with CP. Moreover, His tag pull-down assays confirmed the direct interaction of MmHSP90ab1 with CP (Fig 1D). The interaction between CP and HSP90ab1 was also found in another two marine fish species, *Lateolabrax japonicus* and *Bostrychus sinensi* (Fig 1E). These results strongly indicate that MmHSP90ab1 binds to CP.

### Domain mapping of the association between MmHSP90ab1 and CP

**Fig 2:**
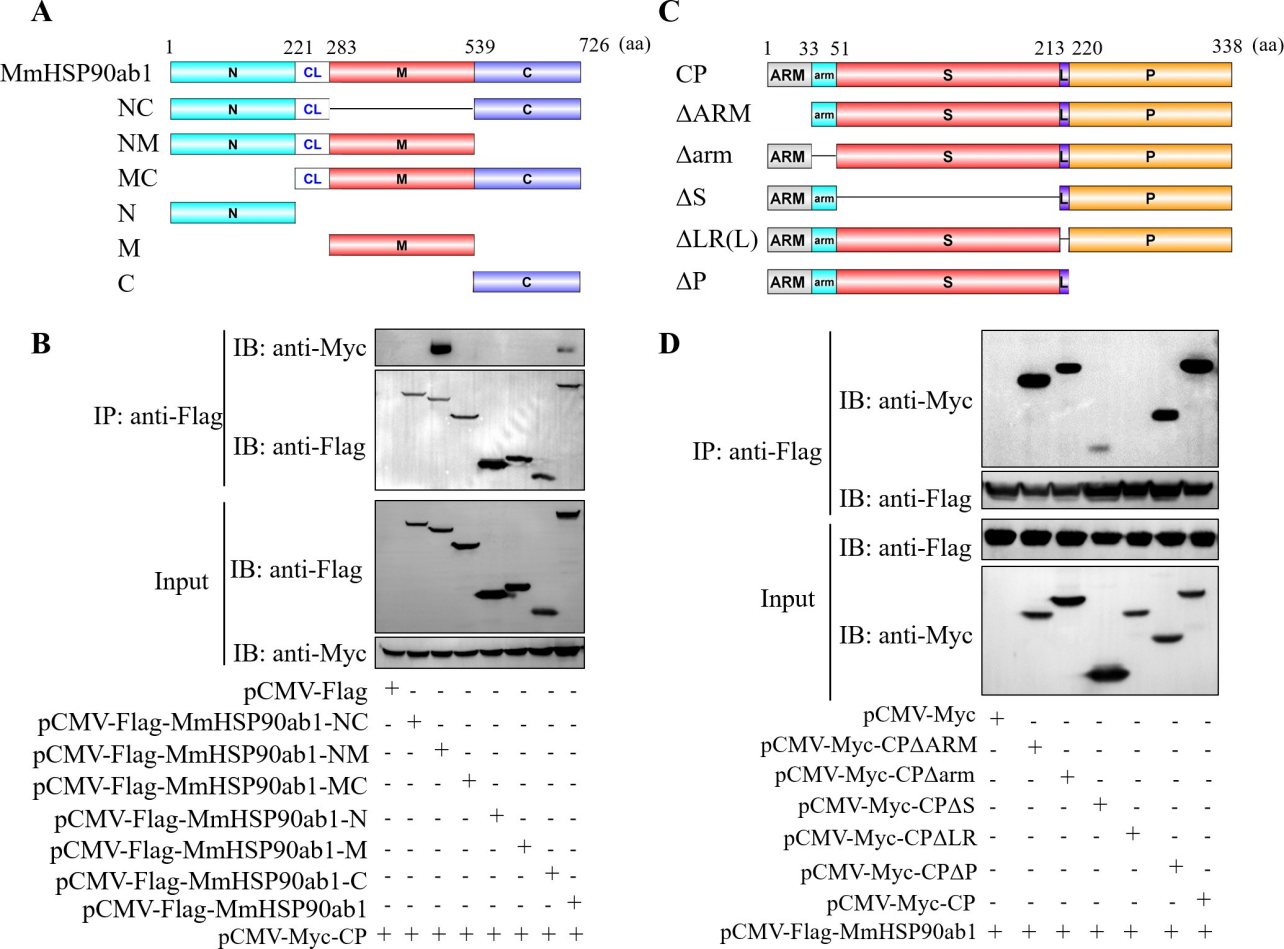
MmHSP90ab1 interacts with the LR domain of CP through its NM Domain. (A) Schematic diagram of MmHSP90ab1 and its truncated mutants. (B) HEK293T cells were cotransfected with *pCMV-Myc-CP* and full-length MmHSP90ab1 or its truncated mutants for 48 h. Cell lysates were immunoprecipitated with anti-Flag abs. The immunoprecipitates and input were immunoblotted with the indicated antibodies. (C) Schematic diagram of CP and its deleted mutants. (D) HEK293T cells were cotransfected with Flag-tagged MmHSP90ab1 and different Myc-tagged CP mutants for 48 h. Co-IP assays were performed as described above.

To determine the domain of MmHSP90ab1 required for interaction with CP, various Flag-tagged distinct domains of MmHSP90ab1 were constructed as shown in Fig 2A. HEK293T cells were cotransfected with *pCMV-Myc-CP* and different MmHSP90 domains recombinant plasmids, respectively, followed by Co-IP assays. pCMV-Flag-MmHSP90ab1-NM was associated with pCMV-Myc-CP (Fig 2B), whereas pCMV-Flag-MmHSP90ab1-NC, pCMV-Flag-MmHSP90ab1-MC, pCMV-Flag-MmHSP90ab1-N, pCMV-Flag-MmHSP90ab1-M, pCMV-Flag-MmHSP90ab1-C lost their abilities to interact with pCMV-Myc-CP (Fig 2B), indicating the NM domain of MmHSP90ab1 is required for its interaction with CP. Next, to further examine which domain of CP is required for interaction with MmHSP90ab1, a series of Myc-tagged CP deletion mutants were constructed (Fig 2C). MmHSP90ab1 was coprecipitated with pCMV-Myc-CP-ΔARM, pCMV-Myc-CP-Δarm, pCMV-Myc-CP-ΔS or pCMV-Myc-CP-ΔP, but not with pCMV-Myc-CP-ΔLR (Fig 2D), suggesting the linker region (LR) of CP was crucial for interaction with MmHSP90ab1. Collectively, these data indicate that MmHSP90ab1 interacts with the LR domain of CP through its NM domain.

### Knockdown or inhibition of MmHSP90ab1 reduces the RGNNV entry

**Fig 3:**
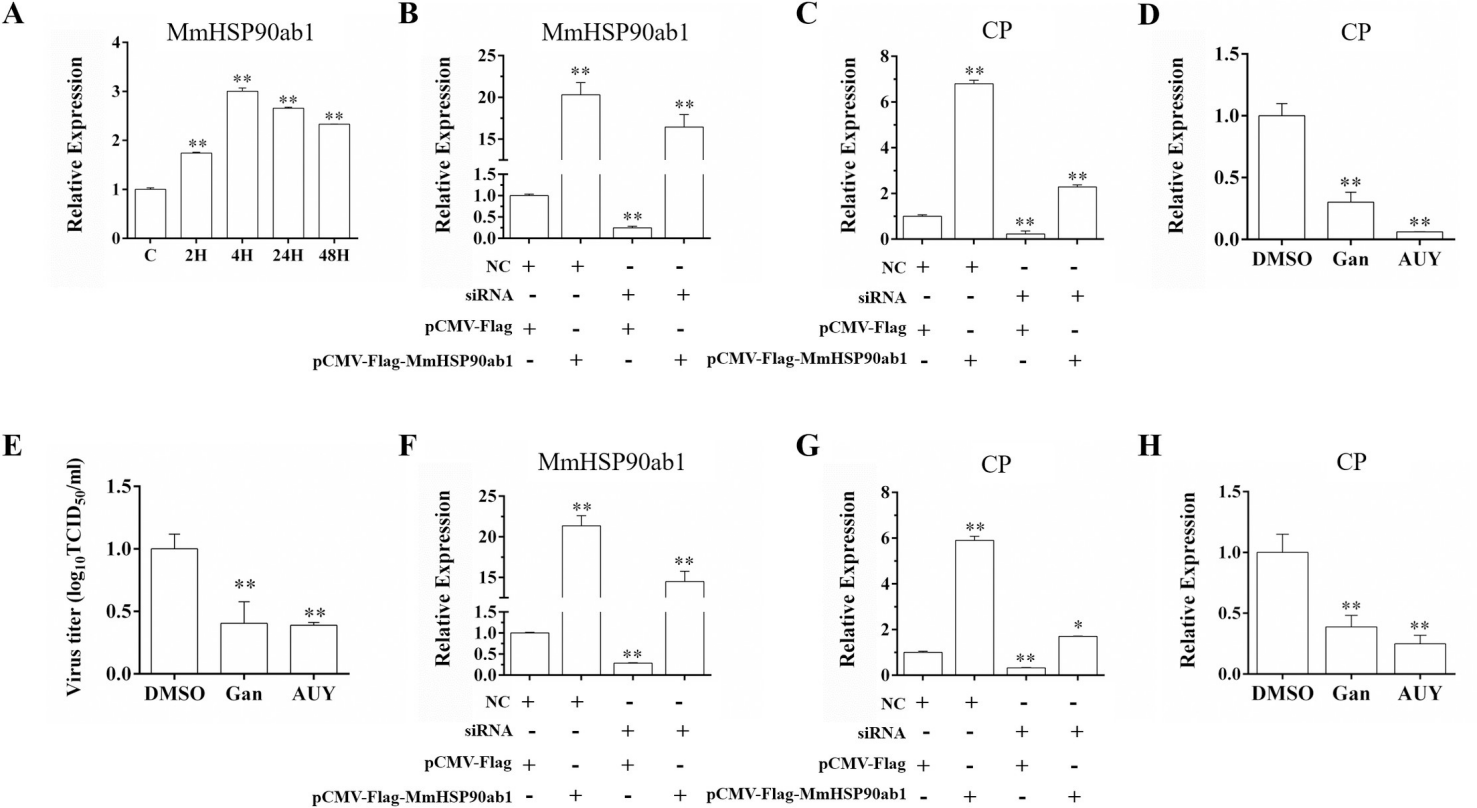
Effect of MmHSP90ab1 on RGNNV entry. (A) Expression analysis of *MmHSP90ab1* in hMMES1 cells at 2, 4, 24 and 48 h post RGNNV infection. (B-E) hMMES1 cells were cotransfected with MmHSP90ab1 siRNA 01 (100 nM) or NC (100 nM) together with *pCMV-Flag* or *pCMV-Flag-MmHSP90ab1* plasmids for 24 h, respectively. Then, cells were infected with RGNNV at 28°C for 4 h (B and C) or at 4°C for 2 h (F and G), the expression of *MmHSP90ab1* (B and F) and the number of viral capsid protein (CP) RNA copies (C and G) were quantified by qRT-PCR. (D and E) hMMES1 cells were treated with ganetespib (Gan) (0.5 μM), AUY922 (AUY) (1 μM), or DMSO (0.1%) for 4 h, then infected with RGNNV at 28°C for 4 h. The number of CP RNA copies and virus titers were measured by qRT-PCR (D) or a plaque assay (E), respectively. (H) hMMES1 cells were treated with ganetespib (Gan) (0.5 μM), AUY922 (AUY) (1 μM), or DMSO (0.1%) for 4 h, then infected with RGNNV at 4°C for 2 h, the amount of attached viral CP RNA copies was quantified by qRT-PCR. The level of *MmHSP90ab1* and the amount of CP RNA copies were normalized to the level of the *β-actin* housekeeping gene. *, (*p* < 0.05); **, (*p* < 0.01).

To determine whether MmHSP90ab1 was involved in RGNNV infection, the expression pattern of *MmHSP90ab1* during RGNNV infection was investigated. As shown in Fig 3A, *MmHSP90ab1* showed a significant highly expression from 2 to 48 hours post infection (hpi), and the highest expression level was observed at 4 hpi, indicating MmHSP90ab1 might play a vital role in the early stages of infection. Thus, we examined the effect of MmHSP90ab1 on RGNNV entry at 28°C. As shown in Fig 3B and 3C, overexpression of MmHSP90ab1 potentiated the entry of RGNNV, whereas knockdown of MmHSP90ab1 by siRNA inhibited RGNNV entry which was restored by the addition of siRNA resistant MmHSP90ab1. Similarly, the treatment of hMMES1 cells with Gan or AUY, the inhibitors of HSP90ab1, significantly decreased RGNNV entry, as suggested by the reduction of CP RNA copies and virus titers in Gan or AUY treated hMMES1 cells (Fig 3D and 3E). Furthermore, we investigated the effect of MmHSP90ab1 on RGNNV binding by treating hMMES1 cells with RGNNV at 4°C for 2 h, on this condition, the virus could bind to cells but not entry into cells [29]. Overexpression of MmHSP90ab1 significantly facilitated RGNNV binding, whereas its knockdown led to the opposite effect which was rescued by overexpression of MmHSP90ab1 (Fig 3F and 3G). Gan or AUY treatment also significantly impaired RGNNV binding (Fig 3H). These results indicate that MmHSP90ab1 participate in RGNNV entry, especially RGNNV binding to hMMES1 cells.

### MmHSP90ab1 localizes on the cell surface

**Fig 4:**
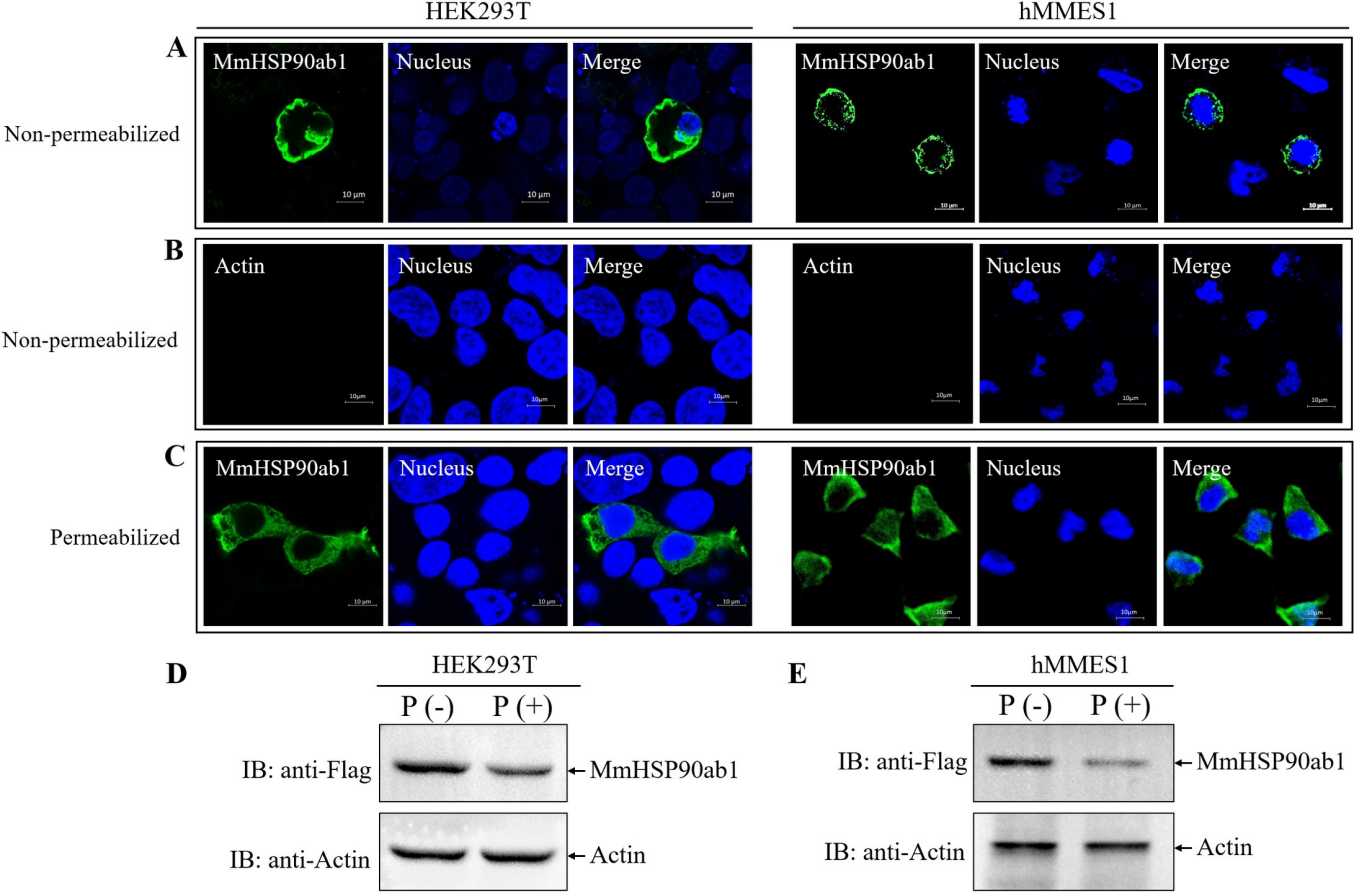
Surface localization of MmHSP90ab1 in HEK293T and hMMES1 cells. (A-B) HEK293T and hMMES1 cells transfected with *pCMV-Flag-MmHSP90ab1* plasmid were fixed with formalin without Triton X-100 and immunostained with anti-Flag abs (A) or anti-Actin abs (B), respectively. (C) HEK293T and hMMES1 cells were transfected with *pCMV-Flag-MmHSP90ab1* plasmid, fixed with formalin and treated with Triton X-100. Finally, cells were immunostained with anti-Flag abs. Cell nuclei were stained with Hoechst 33342. Bar = 10 μm. (D and E) HEK293T and hMMES1 cells transfected with *pCMV-Flag-MmHSP90ab1* plasmid were treated with proteinase K (P+) or without proteinase K (P-) and harvested for a Western blot analysis using anti-Flag and anti-Actin abs.

To further investigate whether MmHSP90ab1 exists on the surface of cells, we transfected *pCMV-Flag-MmHSP90ab1* into HEK293T cells or hMMES1 cells for IF assays using anti-Flag abs under permeabilized or non-permeabilized conditions. As shown in Fig 4, MmHSP90ab1 proteins were observed on the surface of both HEK293T and hMMES1 cells without permeabilization (Fig 4A), meanwhile, no immunofluorescent signal was detected on the surface of non-permeabilized cells using anti-Actin abs (Fig 4B). In contrast, in permeabilized cells treated with Triton X-100, MmHSP90ab1 proteins were found not only on the cell surface but in the cytoplasm (Fig 4C). To further confirm that MmHSP90ab1 is localized on the cell surface, a protease protection assay was carried out. As shown in Fig 4D and 4E, the addition of proteinase K resulted in the significant reduction of MmHSP90ab1 protein in nonpermeabilized HEK293T or hMMES1 cells transfected with *pCMV-Flag-MmHSP90ab1* plasmid compared to the control without proteinase K treatment. These data indicate that MmHSP90ab1 can localize on the surface of cells.

### MmHSP90ab1 protein is a surface receptor of RGNNV

**Fig 5:**
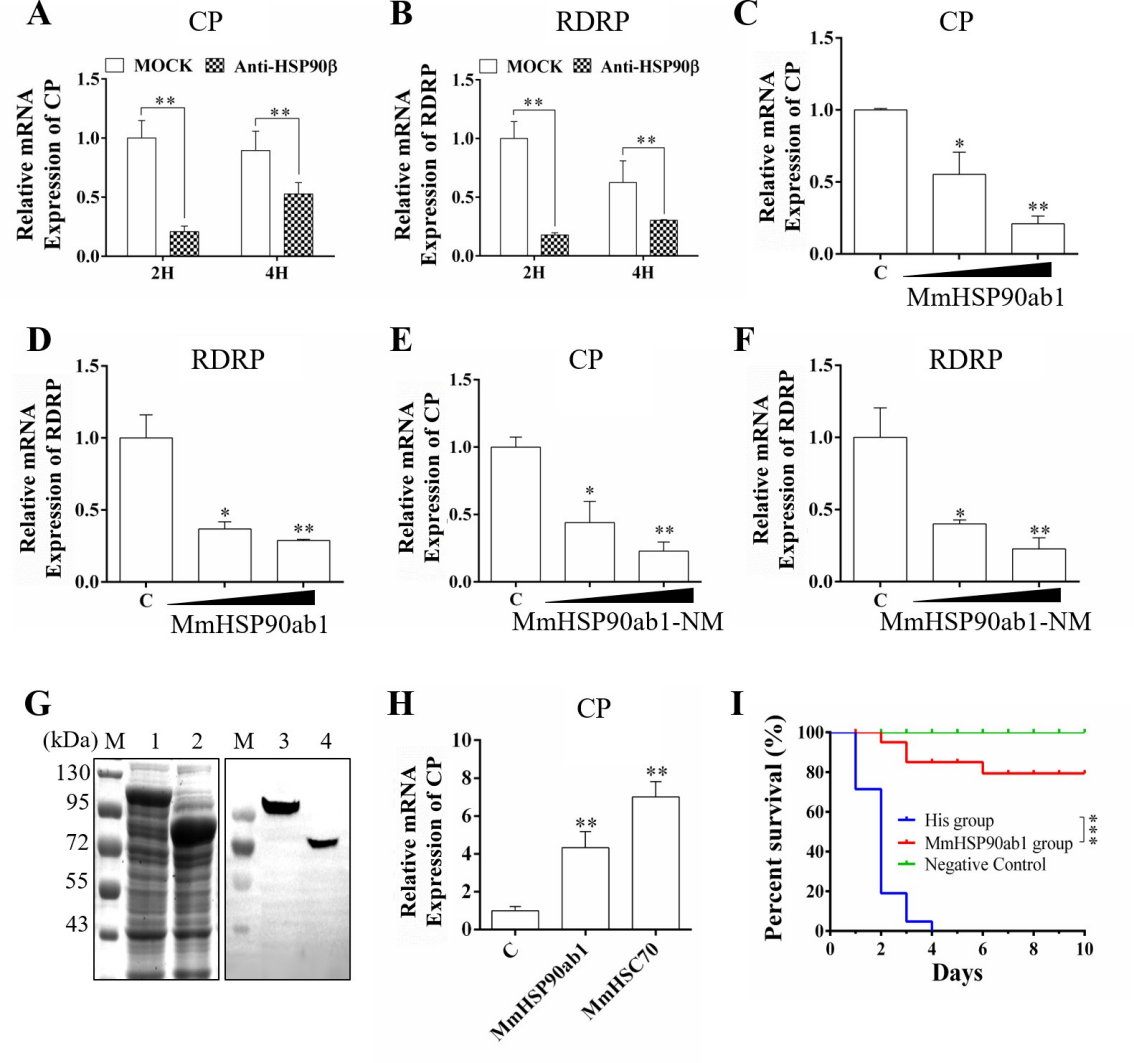
Blocking assay of RGNNV entry. (A and B) hMMES1 cells were incubated with commercial anti-human HSP90β abs (1:50) for 4 h and then infected with RGNNV (MOI = 5) for 2 or 4 h at 4°C. After washed with PBS for three times, cells were harvested for *CP* (A) and *RDRP* (B) expression detection. (C-F) RGNNV was incubated with purified His-MmHSP90ab1 (100 or 500 ng) (C and D) or His-MmHSP90ab1-NM (100 or 500 ng) proteins (E and F) for 4 h at 4°C, then was added to hMMES1 cells which were further incubated for 4 h at 4°C. Cells were washed with PBS for three times and harvested for *CP* (C and E) and *RDRP* (D and F) expression detection. (G) Recombinant expression and purification of His-MmHSP90ab1and His-MmHSP90ab1-NM. Total proteins from *E*. *coli* with His-MmHSP90ab1 (Lane 1) or His-MmHSP90ab1-NM (Lane 2) after IPTG induction; purified recombinant His-MmHSP90ab1 (Lane 3) or His-MmHSP90ab1-NM (Lane 4); lane M, protein marker. (H) HEK293T cells were transfected with *pEGFP-MmHSP90ab1* (MmHSP90ab1), *pEGFP-MmHSC70* (MmHSC70) or *pEGFP-N3* plasmids (C), respectively. Then, transfected cells were infected with RGNNV (MOI = 10) for 4 h at 4°C. Next, the cells were washed to remove any unbound viruses and total RNA was extracted for *CP* detection by qRT-PCR. (I) Survival rates of marine medaka infected with RGNNV and MmHSP90ab1 or His protein mixtures. RGNNV (100 TCID_50_) was mixed with purified His-tagged MmHSP90ab1 recombinant protein or His protein and incubated for 4 h at 4°C. Then fish were intraperitoneally injected with mixtures, respectively. The same volume of PBS was injected as negative control. The cumulative survival rate was determined from 1 to 10 days post-infection. *, (*p* < 0.05); **, (*p* < 0.01), ***, (*p* < 0.001).

To further substantiate that MmHSP90ab1 is an attachment receptor for RGNNV infection, commercial anti-human HSP90β abs and purified His-tagged MmHSP90ab1 or MmHSP90ab1-NM proteins were used to evaluate the role of MmHSP90ab1 in the virus binding process. As shown in Fig 5A and 5B, levels of *CP* and *RDRP* were significantly decreased at 2 and 4 hpi, suggesting that RGNNV attachment was blocked by anti-HSP90β abs. Additionally, recombinant MmHSP90ab1 and MmHSP90ab1-NM proteins significantly reduced the binding of RGNNV to hMMES1 cells in a dose-dependent manner (Fig 5C–5G). Similar with MmHSC70, a known NNV receptor, overexpression of MmHSP90ab1 promoted the attachment of RGNNV to the surface of HEK293T cells (Fig 5H). The marine medaka challenged with MmHSP90ab1 protein and RGNNV mixtures had a relatively higher survival rate compared with that challenged with His and RGNNV mixtures (Fig 5I). Taken together, these results demonstrate that MmHSP90ab1 protein is a surface receptor of RGNNV.

### MmHSC70 interacts with MmHSP90ab1-CP complex

**Fig 6:**
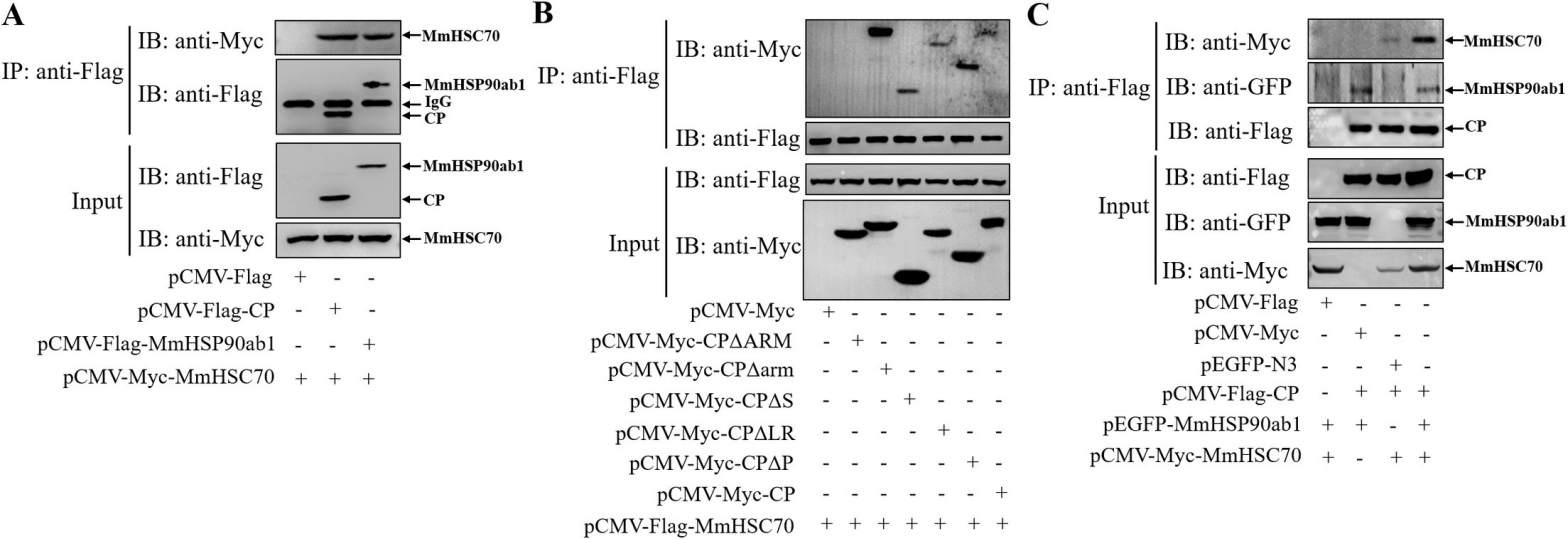
The interactions among MmHSP90ab1, MmHSC70 and CP. (A) HEK293T cells were cotransfected with *pCMV-Myc-MmHSC70* and *pCMV-Flag*, *pCMV-Flag-CP* or *pCMV-Flag-MmHSP90ab1* plasmids for 48 h, respectively. Cell lysates were immunoprecipitated with anti-Flag abs and analyzed by western blotting with anti-Myc and anti-Flag abs. (B) Flag-tagged MmHSC70 and different Myc-tagged CP mutants were cotransfected into HEK293T cells. Co-IP assays were performed as described above. (C) HEK293T cells were cotransfected with indicated expression plasmids for 48 h. Co-IP assays were performed using anti-Flag abs. Cell lysates and immunoprecipitated proteins were analyzed by western blotting using anti-Myc, anti-Flag, and anti-GFP abs.

It has been reported that the grouper HSC70, functioning as an NNV receptor or co-receptor protein, participates in the NNV entry of GF-1 cells by interacting with CP [12]. Thus, we examined whether CP also interacted with marine medaka HSC70 (MmHSC70) through Co-IP assays. CP was shown to coprecipitate with MmHSC70 (Fig 6A). In addition, MmHSP90ab1-MmHSC70 interaction was also found (Fig 6A). To map CP domains involved in CP-MmHSC70 binding, HEK293T cells were transfected with Flag-MmHSC70 and CP deletion mutants. Co-IP assays showed that the ARM domain of CP was responsible for its association with MmHSC70 (Fig 6B). As expected, both MmHSC70 and MmHSP90ab1 proteins were coimmunoprecipitated with CP (Fig 6C), indicating a CP-MmHSC70-MmHSP90ab1 complex was formed.

### MmHSP90ab1 is involved in the internalization of RGNNV

**Fig 7:**
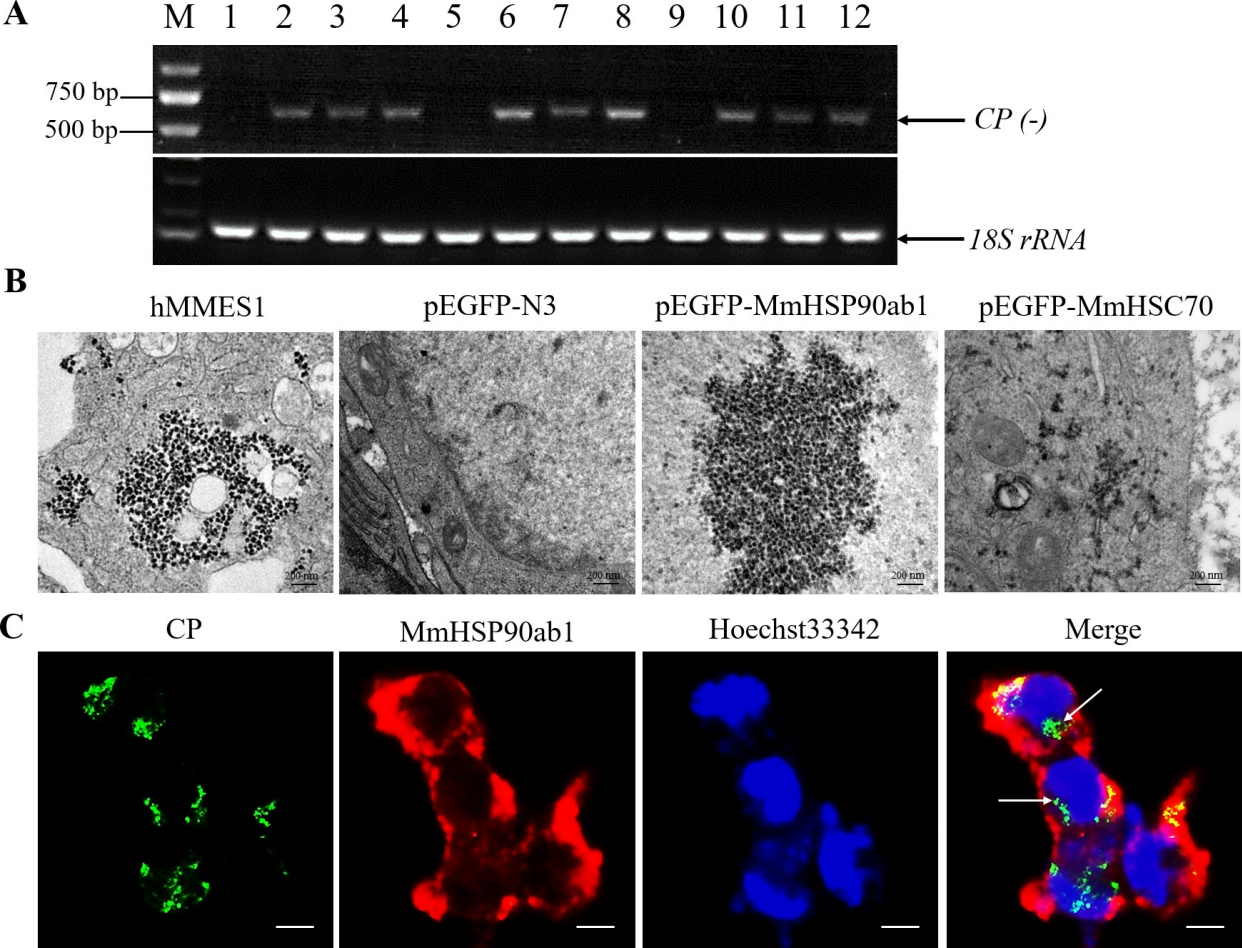
MmHSP90ab1 was involved in RGNNV internalization. (A) RT-PCR analysis of *CP (-)* sequence. HEK293T cells were transfected with *pEGFP-N3* empty vector (1, 5, and 9), *pEGFP-MmHSP90ab1* (2, 6, and 10), *pEGFP-MmHSC70* (3, 7 and 11), or both *pEGFP-MmHSP90ab1* and *pEGFP-MmHSC70* (4, 8, and 12) plasmids for 24 h, respectively. Then cells were infected with RGNNV (MOI = 5) for 4 h. Next, the cells were washed to remove any unbound viruses and incubated for 24 h (1–4), 48 h (5–8) and 72 h (9–12). Cells were harvested and total RNA was extracted for *CP (-)* detection by qRT-PCR. Human *18S rRNA* was detected as reference. (B) Transmission electron micrograph of RGNNV-infected hMMES1 cells and HEK293T cells transfected with *pEGFP-N3* empty vector, *pEGFP-MmHSP90ab1* or *pEGFP-MmHSC70* with 80,000 magnifications. Bar = 200 nm. (C) Immunofluorescence analysis of MmHSP90ab1 and CP proteins. HEK293T cells transfected with *pCMV-Flag* empty vector or *pCMV-Flag-MmHSP90ab1* were infected with RGNNV (MOI = 5) for 24 h, CP (Green) and MmHSP90ab1 (Red) were detected by immunofluorescence staining. Cell nuclei were stained with Hoechst 33342. Bar = 10 μm.

Once viral structural proteins interact with cellular receptors, it will further activate the cellular endocytic pathways to successfully enter host cells. A series of experiments were performed to examine whether MmHSP90ab1 functions in the step of RGNNV internalization. First, the CP anti-reverse sequences (CP (-)), which is a replicative intermediate for production of viral RNAs, were detected in MmHSP90ab1 or MmHSC70-overexpressing HEK293T cells post RGNNV infection, but not in empty vector transfected cells (Fig 7A). Secondly, similar to hMMES1 cells, lots of viral particles were observed in the cytoplasm of MmHSP90ab1 or MmHSC70-overexpressing HEK293T cells post RGNNV infection (Fig 7B). The internalization of RGNNV into the HEK293T-MmHSP90ab1 cells was further evidenced by the intracellular immunofluorescence localization of CP at 24 hpi (Fig 7C). These results suggest that MmHSP90ab1 is not only important for attachment but for internalization.

### MmHSP90ab1-mediated RGNNV internalization is clathrin-dependent

**Fig 8:**
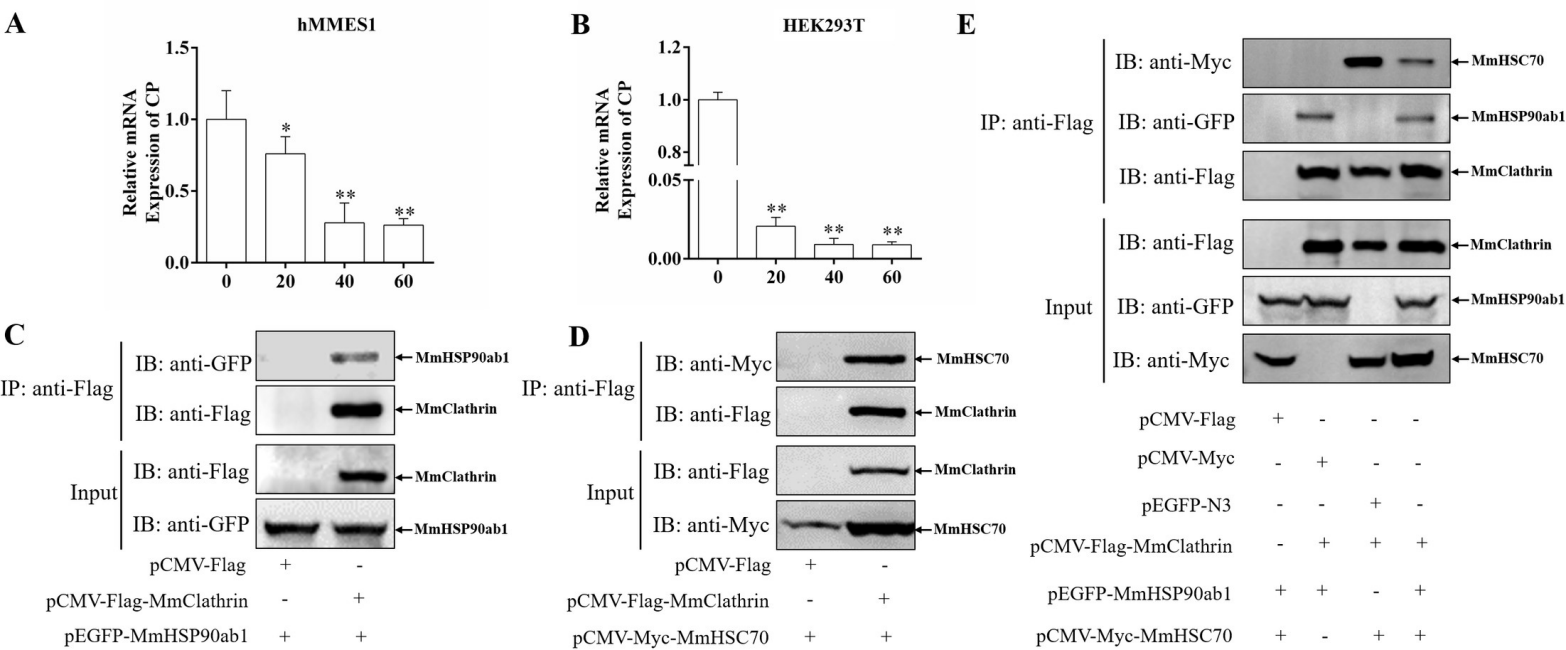
MmHSP90ab1 facilitated RGNNV internalization via clathrin-mediated endocytosis (CME). (A) RGNNV entered into hMMES1 cells through the CME pathway. hMMES1 cells were pretreated with different concentrations of CPZ (0, 20, 40 and 60 μM) for 2 h, then infected with RGNNV (MOI = 10) for 1 h at 4°C. Next, the cells were washed to remove any unbound viruses and incubated at 28°C for 4 h. The expression of *CP* mRNA was detected by qRT-PCR. DMSO was used as control. *, (*P* < 0.05); **, (*P* < 0.01). (B) CME was involved in MmHSP90ab1-mediated RGNNV internalization. HEK293T cells were transfected with *pEGFP-MmHSP90ab1* plasmid, then were treated as described above. The expression of *CP* mRNA was detected by qRT-PCR. **, (*P* < 0.01). (C) MmHSP90ab1 was associated with MmClathrin heavy chain. HEK293T cells were cotransfected with *pEGFP-MmHSP90ab1* and *pCMV-Flag-MmClathrin* or *pCMV-Flag* plasmids for 48 h, respectively. Cell lysates were immunoprecipitated with anti-Flag abs and analyzed by western blotting with anti-GFP and anti-Flag abs. (D) MmHSC70 was associated with MmClathrin heavy chain. *pCMV-Myc-MmHSC70* and *pCMV-Flag-MmClathrin* or *pCMV-Flag* were contransfected into HEK293T cells. Co-IP assays were performed with anti-Flag abs as above. (E) HEK293T cells were cotransfected with indicated expression plasmids for 48 h. Co-IP assays were performed using anti-Flag abs. Cell lysates and immunoprecipitated proteins were analyzed by western blotting using anti-Myc, anti-Flag, and anti-GFP abs.

Clathrin-mediated endocytosis (CME) was previously reported as the primary route of RGNNV internalization, so we wonder whether the MmHSP90ab1-mediated internalization of RGNNV was clathrin-dependent. In the hMMES1 cells treated with CPZ, an effective inhibitor of CME, the amount of internalized RGNNV was significantly reduced in a dose-dependent manner (Fig 8A), demonstrating that RGNNV entered RGNNV-permissive cells via CME. Further studies showed that CPZ dose-dependently reduced MmHSP90ab1-mediated internalization of RGNNV in MmHSP90ab1-overexpressing HEK293T cells (Fig 8B), indicating MmHSP90ab1-mediated internalization was clathrin-dependent. In addition, Co-IP showed that MmHSP90ab1 and MmHSC70 were associated with marine medaka Clathrin (MmClathrin), synchronously or respectively (Fig 8C–8E). Taken together, these results suggest that MmHSP90ab1 facilitate RGNNV internalization through CME.

## Discussion

Virus receptors are pivotal for virus host tropism [30, 31]. Although NNV has a wide host tropism and can infect more than 120 marine and freshwater fish species [2], to date, only HSC70 was identified as an attachment receptor for NNV [12]. Thus, it is still an important and urgent task to identify NNV receptors or co-receptors and clarify their mechanisms of action. In the present study, MmHSP90ab1 was identified as a functional component of receptor complexes for RGNNV for the first time.

HSP90ab1, also known as HSP90β, is a member of the HSP90 family that interacts with a set of co-chaperones and plays essential roles in various cellular processes [32]. Here, utilizing an IP assay followed by MS, we identified lots of potential proteins interacting with CP, and the interaction between MmHSP90ab1 and CP was further reconfirmed by co-focusing, Co-IP and pull-down assays. Furthermore, we found that the interaction of HSP90ab1 with CP was conserved across fish species which were susceptible to NNV, indicating HSP90ab1 might play important roles in RGNNV infection. Structurally, our results showed that the NM domain (containing regions NTD, CL, and MD) of MmHSP90ab1 was required for its interaction with CP. However, the CL region, which is important for HSP90 client protein folding [33], was not necessary for MmHSP90ab1-CP interaction (S1 Fig). All these results indicate that targeting MD and NTD of MmHSP90ab1 may be an effective approach for interfering RGNNV infection. For the distinct regions of CP as described by Chen et al [34], we further found the LR domain of CP was critical for its interaction with MmHSP90ab1. Further detailed studies are needed to determine the exact amino acid of MmHSP90ab1 in NM that is responsible for MmHSP90ab1-CP interaction.

Here, the binding and entry of RGNNV were significantly inhibited not only by MmHSP90ab1 special siRNA but HSP90 inhibitors Gan and AUY in hMMES1 cells, suggesting that MmHSP90ab1 plays an important role in RGNNV binding and entry. Previously, multiple studies have reported that HSP90β was a cellular receptor for Japanese encephalitis virus [20], dengue virus [26], and enterovirus 71 [35]. Considering the interaction of CP and MmHSP90ab1 and its involvement with RGNNV binding, we speculated that MmHSP90ab1 might be a receptor for RGNNV. To verify the hypothesis, a series of experiments were carried out. First, IF assays carried out on non-permeabilized cells revealed that MmHSP90ab1 proteins were expressed on the cell surface of HEK293T and hMMES1 cells, which was consistent with the increasing evidence that HSP90β proteins were not only localized in the cytoplasm but also on the cell surface [21, 26, 36]. Secondly, we found that anti-human HSP90β abs significantly inhibited RGNNV binding which further supported the conclusion that the N-terminus domain of MmHSP90ab1, recognized by anti-human HSP90β abs, was vital for CP-MmHSP90ab1 interaction. The fact that recombinant MmHSP90ab1 and MmHSP90ab1-NM proteins blocked RGNNV entry in a dose-dependent manner was probably another piece of evidence to indicate that MmHSP90ab1 was a receptor for RGNNV in hMMES1 cells. In addition, the results of animal experiments showed that MmHSP90ab1 protein partially protected marine medaka from RGNNV infection. These results supported the conclusion of MmHSP90ab1 as a novel functional receptor for RGNNV infection. Considering interactions of CP and HSP90ab1 of *O*. *melastigma*, *L*. *japonicus* and *B*. *sinensis*, we speculated that HSP90ab1 might be a universal receptor for RGNNV in fish.

The virus entry is a complex process, in which the host receptors may not work alone [19]. Previously, grouper HSC70 has been identified as a potential receptor or co-receptor interacting with CP in the early stages of grouper NNV (GNNV) infection [12]. Herein, we also reported the interaction of CP and MmHSC70, indicating this interaction was conservative among different fish species. The ARM domain of CP is thought to play an important role in virus particle assembly [34]. Here, CP ARM was identified as a critical region for the interaction between CP and MmHSC70, suggesting ARM might have novel molecular function. As expect, CP, MmHSP90ab1, and MmHSC70 formed a complex, therefore, it is reasonable to assume that NNV can simultaneously use MmHSP90ab1 and MmHSC70 as receptors for its entry. The P domain of CP is a major distinct region between different genotypes of the genus *Betanodavirus* [37]. It has been reported that a PEG-binding site on the P domain of GNNV CP seems to favor GNNV infection, several hypervariable regions on the P domain are good candidates for host specificity determinants [28, 38], implying the participation of some other unidentified receptor(s) interacting with CP P domain during RGNNV infection.

**Fig 9:**
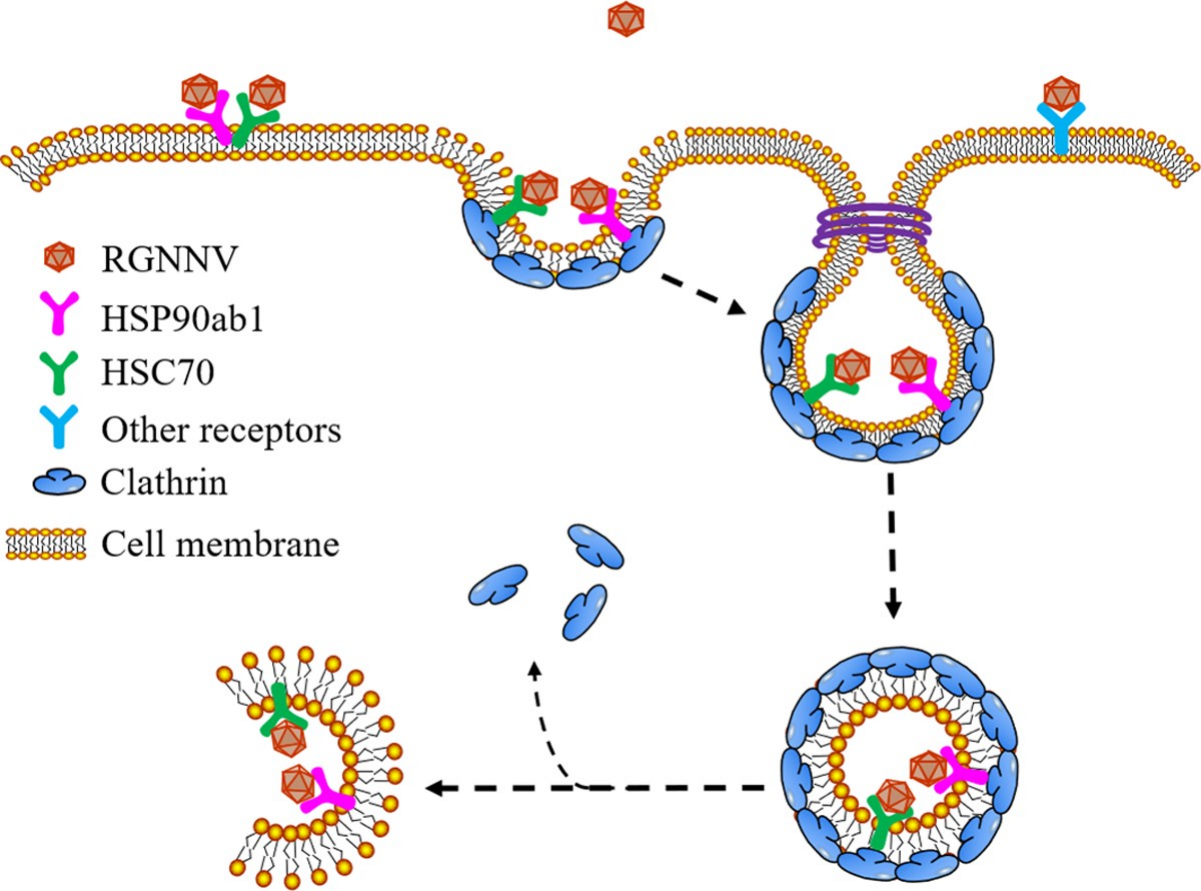
Model of the role of MmHSP90ab1 in RGNNV entry into host cells. During infection, RGNNV might attach to cells by the interaction between CP and HSP90ab1, HSC70 or other receptor(s) on the cell surface. Then, CP, its receptor(s), and clathrin formed a protein complex that facilitated RGNNV internalization through the clathrin-mediated endocytosis pathway.

Previous study demonstrated that RGNNV could attach to HEK293T cells and replicate in HEK293T cells transfected with viral RNA, but could not penetrate them [39]. Here, we found that overexpression of MmHSP90ab1 not only promoted the binding of RGNNV to HEK293T cells, but also RGNNV internalization, as indicated by the detection of CP anti-reverse sequence, a replication form of viral RNAs [40], CP proteins and lots of viral particles in MmHSP90ab1-overexpressing HEK293T cells. All these findings indicate that MmHSP90ab1 can facilitate RGNNV internalization independently. The CME pathway is the common endocytosis pathway utilized by all kinds of viruses to enter the host cells. Several studies have demonstrated that the principal route of RGNNV into host cells is the CME pathway [41]. HSC70 and its co-chaperones were known to be involved in and played vital roles in the CME pathway [42, 43]. In the present study, we found that MmHSP90ab1 aided in RGNNV internalization through the CME pathway. Meanwhile, MmHSP90ab1 and MmHSC70 both associated with MmClathrin heavy chain and formed a complex of MmHSP90ab1-MmClathrin-MmHSC70. Topologically, HSP90 and HSC70 are cytosolic proteins, while increasing evidence reveals the presence of HSP90 and HSC70 on the cell surface in recent years [44–46]. Such surface exposed HSP90 and HSC70 proteins participate in the viral entry by functioning as receptor or co-receptor of multiple viruses, such as dengue virus, enterovirus 71 and rotavirus [26, 35, 47]. Therefore, it is reasonable to speculate that RGNNV may bind to surface exposed HSP90ab1 and HSC70 alone or together, or the other unidentified receptors on the cell surface, then, CP, its receptors, and clathrin form a protein complex which facilitates RGNNV enter host cells through the CME pathway (Fig 9). Future work will be necessary to further define the physiological roles of HSC70 and HSP90ab1 during CME.

In addition to functioning as a virus receptor or co-receptor on the cell surface, multiple studies have reported that HSP90ab1 also plays crucial roles in different phases of the virus life cycle, such as viral assembly, replication, and nuclear translocation. For instance, HSP90β (HSP90ab1) enhanced enterovirus 71 viral particles assembly [21]. HSP90ab1 rescued ritonavir-resistant HIV replication [48]. HSP90ab1 was incorporated into HIV virions and could rescue the infectivity of HIV with defective cores [49]. Hsp90β (HSP90ab1) facilitated the nuclear transfer of Epstein-Barr virus DNA polymerase [50]. Thus, further experiments directed to analyze the intracellular signaling induced by MmHSP90ab1 and CP interaction and the multifactorial role of MmHSP90ab1 in other stages of RGNNV replication cycle are being performed in our laboratory.

In summary, MmHSP90ab1 was identified as a novel functional attachment receptor for RGNNV. Moreover, MmHSP90ab1 was involved in and facilitated RGNNV internalization through the CME pathway. Our findings would be beneficial to extend our understanding of the host cell response and pathogenesis mechanism of RGNNV and shed a new insight into the development of novel antiviral therapies.

## Material and methods

### Animal ethic statements

All procedures with marine medaka were approved by the Ethics Committee of Sun Yat-Sen University and the methods were carried out following the approved guidelines.

### Cells, virus, and reagents

hMMES1 cell line was established from marine medaka embryo blastocysts and was susceptible to RGNNV. hMMES1 cells were cultivated in ESM4 medium at 28°C as previously described [51]. HEK293T cells were cultured in DMEM medium supplemented with 10% heat-inactivated fetal bovine serum (FBS, Gibco, Invitrogen) and incubated at 37°C with 5% CO_2_.

RGNNV originally was isolated from diseased sea perch larvae and juveniles in Guangdong Province of China and proliferated in LJB cells [52, 53]. Virus stocks were stored at −80°C for use.

Anti-Flag (M20008), anti-Myc (M20002), anti-Actin (P30002), and anti-His abs (M20001L) were purchased from Abmart (Guangzhou, China); Anti-human HSP90β abs (ab236282) were purchased from Abcam; Anti-GFP abs (G1544) were purchased from Sigma (St. Louis, MO, USA). The secondary antibody goat anti-rabbit IgG-HRP was purchased from Cell Signaling Technology (Danvers, MA, USA); Alexa Fluor 488-labeled donkey anti-rabbit IgG and Alexa Fluor 555-labeled goat anti-mouse IgG secondary abs, Hoechst 33342, and phenylmethylsulfonyl fluoride (PMSF) were purchased from Invitrogen Corporation (Carlsbad, CA, USA). Proteinase K (1245680100) was purchased from Solarbio (Beijing, China). Triton X-100 was obtained from Sigma-Aldrich. Pharmaceutical grade ganetespib (Gan) and NVP-AUY922 (AUY) were purchased from Beyotime (Guangzhou, China) and prepared as a 10 mM stock in dimethyl sulfoxide (DMSO).

### Plasmid construction

Plasmids expressing full-length MmHSP90ab1 (GenBank accession number XM_024290541.1) were amplified by PCR and cloned into *pCMV-Flag* (Clontech) and *pEGFP-N3* vectors (Clontech), respectively. Coding regions of RGNNV CP (KP455642) were cloned into *pCMV-Flag*, *pCMV-Myc* (Clontech), *pEGFP-N3*, and *pET-32a (+)* (Clontech) vectors, respectively. Clathrin heavy chain of marine medaka (Ensomet00000024422.1) was cloned into *pCMV-Flag*. MmHSP90ab1 and CP truncations were constructed using standard molecular biology techniques. MmHSP90ab1 and MmHSP90ab1-NM was cloned into *pET-32a (+)* vectors, respectively. The ORFs of HSP90ab1 of *L*. *japonicus* (LjHSP90ab1) and *B*. *sinensis* (BsHSP90ab1) were cloned into the *pCMV-Flag* vector to generate plasmids *pCMV-Flag-LjHSP90ab1* and *pCMV-Flag-BsHSP90ab1*, respectively. Primers are listed in S2 Table. All plasmid constructs were examined and confirmed via DNA sequencing.

### Immunoprecipitation (IP) screen assays and SDS-PAGE

IP experiments were performed as described previously with some modifications [54]. hMMES1 cells were transfected with *pEGFP-N3* or *pEGFP-CP* plasmids using Lipofectamine 3000 (Invitrogen), respectively. At 48 h after transfection, cells were lysed with lysis buffer (20mM Tris [pH7.5], 150mM NaCl, 1% Triton X-100, sodium pyrophosphate, β-glycerophosphate, EDTA, Na_3_VO_4_, leupeptin and PMSF) on ice for 30 min. Protein A/G magnetic beads (MCE) were prepared with anti-GFP abs in a rotation wheel for 2 h. The cell lysates were then centrifuged at 12,000g for 15 min. The supernatants were preabsorbed into beads with anti-GFP abs. After incubation at 4°C overnight, beads were washed five times with 1ml of wash buffer on a roller for 5 min each time, followed by centrifugation at 2,000g at 4°C for 3 min. The final pellets for pEGFP-N3 or pEGFP-CP were analyzed through 10% SDS-PAGE and protein bands in the gel were stained with Coomassie brilliant blue.

### Mass spectrometry (MS) analysis

Specific protein bands of the IP immunocomplex (pEGFP-CP) on SDS-PAGE gel were cut and processed for liquid chromatography-tandem mass spectrometry (LC-MS/MS) (Probability-based protein identification by searching sequence databases using mass spectrometry data). The coding region of each protein was identified by blasting against National Center for Biotechnology Information (NCBI) protein database and expressed sequence tag (EST) sequences by using Mascot Server (Matrix Science). Ions score was -10*Log (P), where P means the probability of the randomly observed match. Individual ion scores >19 indicated identity or extensive homology (*P* < 0.05). Only the top-ranked peptide matches were taken into consideration for protein identification.

### Co-IP assays

Co-IP assays were performed as described previously [54]. HEK293T cells in 25-cm^2^ dishes were cotransfected with 10 μg of different plasmid combinations as indicated. At 24 h post-transfection, cells were washed twice with 10 ml ice-cold PBS and lysed in 300 μl lysis buffer (20mM Tris [pH7.5], 150mM NaCl, 1% Triton X-100, sodium pyrophosphate, β-glycerophosphate, EDTA, Na_3_VO_4_, leupeptin and PMSF) at 4°C for 1 h on a rocker platform. Protein A/G magnetic beads were prepared with anti-Flag or anti-Myc abs in a rotation wheel for 2 h. The cell lysates were then centrifuged at 12,000g for 15 min and the supernatants were preabsorbed into beads with indicated abs overnight at 4°C with constant agitation. The beads were washed five times with 1 ml of wash buffer on a roller for 5 min every time, followed by centrifugation at 2,000g at 4°C for 3 min. Final immunoprecipitates and the whole cell lysates were resuspended in SDS loading buffer and analyzed by immunoblotting (IB) using indicated abs.

### His fusion protein expression and pull-down assays

For bacterial expression of His-CP fusion proteins, *pET-32a (+)-CP* plasmids were transformed into *E*. *coli* BL21(DE3) which was cultured in 50 ml of LB medium containing 0.5 mM isopropyl-1-thio-*β*-D-galactopyranoside (IPTG) at 18°C overnight with agitation at 120 rpm. Then, cells were pelleted by centrifugation and lysed in lysis buffer (100 mM Sodium-Phosphate [pH 8.0], 600 mM NaCl, 0.02% Tween-20) via sonication on ice. After centrifugation at 15,000g at 4°C for 20 min, the lysate supernatant containing His-tagged proteins was affinity-purified with Dynabead His-Tag magnetic beads (Invitrogen) and used for pull-down assays.

His pull-down assays were performed as described previously with some modifications [54]. His-CP-magnetic beads were washed three times with lysis buffer to remove unbound His-CP and were used to bind Flag-tagged protein from the lysates of HEK293T cells transfected with *pCMV-Flag-MmHSP90ab1* or *pCMV-Flag* empty vectors, respectively. After incubation at 4°C overnight, the beads were washed and analyzed via immunoblot analysis using anti-Flag abs to detect Flag-MmHSP90ab1 proteins. His alone was also prepared and served as a negative control.

### Immunoblot analysis

IB assays were performed as described previously [54]. Immunoprecipitates or whole cell lysates were separated on a 10% SDS-PAGE gel and transferred onto polyvinylidene difluoride (PVDF) membranes (Bio-Rad Laboratories) that were subsequently blocked with TBST buffer (25 mM Tris-HCl, 150 mM NaCl, 0.1% Tween-20 [pH 7.5]) containing 5% nonfat dried milk for 1 h at room temperature (RT). Thereafter, the membranes were probed with the indicated primary abs in an appropriate dilution at 4°C overnight. Following three times wash with TBST, the membranes were further incubated with secondary abs for 1 h at RT. Immunoreactive bands were visualized after three additional washes with TBST buffer.

### RGNNV infection and RNAi

hMMES1 cells were seeded into 24-well plates at 3×10^4^ cells per well and infected with RGNNV (MOI = 1) for 2, 4, 24, and 48 h, respectively. At different time points, cells were harvested for RNA isolation using the RNA extraction kit (Takara) according to the manufacturer’s instructions.

Three short interfering RNAs (siRNAs) targeting MmHSP90ab1 were designed by Ribobio Company (Guangzhou, China). Sequences of siRNAs against *MmHSP90ab1* mRNA were as follows: siRNA 01 (5’-CTACTACATCACTGGTGAA-3’), siRNA 02 (5’-GAAGACCAAACAGAGTACA-3’), and siRNA 03 (5’-TCGACATCATCCCTAACAA-3’). A control siRNA (NC) that has no homology with *MmHSP90ab1* mRNA was used as a control. hMMES1 cells were transfected separately with MmHSP90 siRNA or NC together with *pCMV-Flag* or *pCMV-Flag-MmHSP90ab1* plasmids using Lipofectamine 3000 according to the instructions of the manufacturer. Twenty-four hours after transfection, cells were infected with RGNNV (MOI = 5) for 4 h at 28°C for virus entry or for 2 h at 4°C for virus binding, respectively. Total RNA of cells was extracted for quantitative real-time polymerase chain reaction (qRT-PCR).

### Gan and AUY922 treatment

For MmHSP90ab1 inhibition experiments, hMMES1 cells were pretreated with Gan (0.5 μM) or AUY922 (1 μM) for 4 h prior to RGNNV infection at 28°C for 4 h for virus entry or at 4°C for 2 h for virus binding, respectively. Cells treated in parallel with DMSO served as controls. Total RNA of cells was extracted for viral RNA analyses.

To detect the viral titers, hMMES1 cells pretreated with Gan or AUY922 were infected with RGNNV at 28°C for 4 h, the culture medium was removed and incubated with fresh medium for 48 h, then the supernatant was collected for viral titer assay as described previously [52].

### Immunofluorescence (IF) assays

To examine the localization of MmHSP90ab1 proteins on cells, IF assays were performed as described previously with some modification [54]. Briefly, hMMES1 and HEK293T cells were seeded into 12-well culture plates on glass coverslips and were separately transfected with *pCMV-Flag* and *pCMV-Flag-MmHSP90ab1* plasmids, respectively. After transfection for 36 h, cells were washed with PBS, fixed with 4% paraformaldehyde for 10 min at RT. One group was treated with 0.2% Triton X-100 for membrane permeabilization, and the other was not. After being washed three times with PBS, cells were blocked with PBS containing 5% bovine serum albumin at RT for 1 h and then reacted with anti-Flag abs (1:200) at 4°C overnight. Anti-Actin abs were used as negative control. After three times wash with PBS, cells were incubated with Alexa Fluor 488 donkey anti-rabbit IgG (Invitrogen) at a dilution of 1:100 for 1 h at RT. Cells were then washed with PBS and stained the cell nuclei with Hoechst 33342 for 10 min. Finally, cells were observed under a confocal microscope (LSM510; Zeiss, Germany).

For assessment of the colocalization of MmHSP90ab1 and CP, HEK293T cells were cotransfected with *pCMV-Flag-MmHSP90ab1* and *pCMV-Myc-CP* plasmids. After transfection for 36 h, cells were washed with PBS then fixed and permeabilized as described above. The cells were incubated with both mouse anti-Myc and rabbit anti-Flag abs at a dilution of 1:200 at 4°C overnight, and then detected with Alexa Fluor 555 goat anti-mouse IgG (Invitrogen) and Alexa Fluor 488 donkey anti-rabbit IgG (Invitrogen). Samples were viewed and evaluated by confocal microscopy.

For RGNNV entry detection, HEK293T were transfected with *pEGFP-MmHSP90ab1* for 24 h, and then infected with RGNNV. After infection for 24 h, the cells were detected with IF assays, cells were incubated with rabbit anti-CP abs and detected as described above.

### Proteinase K protection assay

The proteinase K protection assay was performed as described previously with some modification [55]. Briefly, hMMES1 and HEK293T cells were seeded into 6-well culture plates and transfected with *pCMV-Flag-MmHSP90ab1* plasmids for 24 h. After washed with PBS for three times, one group of cells were treated with 10 μg/ml of proteinase K for 30 min in an ice-water bath, another group was added with the same volume of PBS, the reaction was stopped by addition of PMSF. Cells were then lysed with lysis buffer on ice for 30 min, the whole cell lysates were resuspended in SDS loading buffer and analyzed by Western blot using anti-Flag or anti-Actin abs.

### Blocking assays

hMMES1 cells were pre-seeded in 24-well plates overnight. Due to the unavailability of anti-MmHSP90ab1 abs and the high homogeneity of HSP90β (HSP90ab1) at the N-terminus domain between human and fish, cells were incubated with anti-human HSP90β abs (1:50) (Invitrogen) for 3 h at 28°C. After washed with fresh media, cells were infected with RGNNV (MOI = 5) at 4°C for 2 and 4 h, respectively. As a control, hMMES1 cells were in parallel pretreated with normal rabbit IgG. Cells were then washed three times with PBS to remove free virus particles and harvested for total RNA extraction and qRT-PCR detection of RGNNV *CP* and *RDRP*.

His-MmHSP90ab1 and His-MmHSP90ab1-NM proteins were affinity-purified as described above. RGNNV (10^3^ TCID_50_) were incubated with different concentrations (100 or 500 ng) of recombinant MmHSP90ab1 or recombinant MmHSP90ab1-NM proteins for 4 h at 4°C. Then, hMMES1 cells were incubated with virus and protein mixtures for 4 h at 4°C. Similarly, cells were treated with 10^3^ TCID_50_ of RGNNV preincubated with BSA (500 ng) as a control. Then, cells were harvested, *CP* and *RDRP* mRNA were measured as described above.

### qRT-PCR analysis

qRT-PCR was performed in a LightCycler 480 Ⅱ thermal cycler (Roche Applied Science, Germany) with the cycling conditions of 95°C for 30 s, 45 cycles of 95°C for 15 s, 60°C for 15 s, and 72°C for 15 s followed by the melting curve analysis to verify the specificity of amplified products. Primer sequences are listed in S2 Table. For each sample, triplicated experiments were performed and mean Cq values were derived and calculated through the ΔΔCt method [56]. Marine medaka *β-actin* was used as the reference.

### Transmission electron microscopy (TEM)

Cell samples and ultrathin sections for TEM were prepared as described previously [57]. Cells were collected and fixed at 4°C for 24 h with 2.5% glutaraldehyde in 0.1 M PBS (pH 7.4) and 2.0% osmium tetroxide in 0.1 M PBS in turn, ultrathin sections were observed under a Philips CM10 transmission electron microscope.

### Survival assay

The survival rate was calculated in healthy marine medaka. Marine medaka were divided into two groups: the MmHSP90ab1 group and the His group (n = 30). Correspondingly, RGNNV (100 TCID_50_) was incubated with purified His-tagged MmHSP90ab1 recombinant protein or His protein for 4 h at 4°C, then the mixtures were injected into two groups of marine medaka separately. The negative control group of fishes were injected with the same volume of PBS. The survival rate of each group was recorded every day by counting the numbers of dead marine medaka. The log-rank test method was used to analyze the differences between groups. *p* < 0.001, ***.

### Clathrin inhibitor assay

hMMES1 cells or MmHSP90ab1-overexpressing HEK293T cells were treated with different concentrations (20, 40, and 60 μM) of CPZ for 2 h, then infected with RGNNV (MOI = 10) for 1 h at 4°C. Next, the cells were washed to remove any unbound viruses and incubated at 28°C for 4 h. DMSO was used as control. Total RNA of cells was extracted for qRT-PCR detection.

### Statistical analysis

All statistics from qRT-PCR detection in this study were carried out using SPSS version 20. One-way ANOVA was used to determine the differences between groups. *p* < 0.05 was considered to be statistically significant and *p* < 0.01 was considered highly significant.

## Supporting information

S1 Table Summary of CP-interacting proteins identified by Co-IP assays followed by MS. (XLSX)

S2 Table Primers used in this study. (XLSX)

S1 Fig The CL region of MmHSP90ab1 was not necessary for MmHSP90ab1-CP interaction. HEK293T cells were cotransfected with *pCMV-Myc-CP* and *pCMV-Flag-MmHSP90ab1-NM* or *pCMV-Flag-MmHSP90ab1-NMΔ CL* for 48 h, respectively. Cell lysates were immunoprecipitated with anti-Flag abs. The immunoprecipitates and input were immunoblotted with the indicated abs.
(TIF)

## References

[1] Toffan A, Pascoli F, Pretto T, Panzarin V, Abbadi M, Buratin A, et al Viral nervous necrosis in gilthead sea bream (Sparus aurata) caused by reassortant betanodavirus RGNNV/SJNNV: an emerging threat for Mediterranean aquaculture. Sci Rep. 2017;7:46755 10.1038/srep46755 28462930 PMC5411978

[2] Costa JZ, Thompson KD. Understanding the interaction between Betanodavirus and its host for the development of prophylactic measures for viral encephalopathy and retinopathy. Fish Shellfish Immunol. 2016;53:35–49. 10.1016/j.fsi.2016.03.033 . 26997200

[3] Mori K, Nakai T, Muroga K, Arimoto M, Mushiake K, Furusawa I. Properties of a new virus belonging to nodaviridae found in larval striped jack (Pseudocaranx dentex) with nervous necrosis. Virology. 1992;187(1):368–71. Epub 1992/03/01. 10.1016/0042-6822(92)90329-n . 1736540

[4] Toffolo V, Negrisolo E, Maltese C, Bovo G, Belvedere P, Colombo L, et al Phylogeny of betanodaviruses and molecular evolution of their RNA polymerase and coat proteins. Mol Phylogenet Evol. 2007;43(1):298–308. 10.1016/j.ympev.2006.08.003 PubMed PMID: WOS:000245936000022. 16990016

[5] Nishizawa T, Mori K, Furuhashi M, Nakai T, Furusawa I, Muroga K. Comparison of the coat protein genes of five fish nodaviruses, the causative agents of viral nervous necrosis in marine fish. J Gen Virol. 1995;76 (Pt 7):1563–9. Epub 1995/07/01. 10.1099/0022-1317-76-7-1563 . 9049363

[6] Skliris GP, Krondiris JV, Sideris DC, Shinn AP, Starkey WG, Richards RH. Phylogenetic and antigenic characterization of new fish nodavirus isolates from Europe and Asia. Virus Res. 2001;75(1):59–67. Epub 2001/04/20. 10.1016/s0168-1702(01)00225-8 . 11311428

[7] Boulant S, Stanifer M, Lozach PY. Dynamics of virus-receptor interactions in virus binding, signaling, and endocytosis. Viruses. 2015;7(6):2794–815. Epub 2015/06/05. 10.3390/v7062747 26043381 PMC4488714

[8] Zhang Q, Yoo D. PRRS virus receptors and their role for pathogenesis. Vet Microbiol. 2015;177(3–4):229–41. Epub 2015/04/29. 10.1016/j.vetmic.2015.04.002 . 25912022

[9] Hu B, Zhang Y, Jia L, Wu H, Fan C, Sun Y, et al Binding of the pathogen receptor HSP90AA1 to avibirnavirus VP2 induces autophagy by inactivating the AKT-MTOR pathway. Autophagy. 2015;11(3):503–15. Epub 2015/02/26. 10.1080/15548627.2015.1017184 25714412 PMC4502722

[10] Liu Q, Bradel-Tretheway B, Monreal AI, Saludes JP, Lu X, Nicola AV, et al Nipah virus attachment glycoprotein stalk C-terminal region links receptor binding to fusion triggering. J Virol. 2015;89(3):1838–50. Epub 2014/11/28. 10.1128/JVI.02277-14 25428863 PMC4300768

[11] Liu W, Hsu CH, Hong YR, Wu SC, Wang CH, Wu YM, et al Early endocytosis pathways in SSN-1 cells infected by dragon grouper nervous necrosis virus. J Gen Virol. 2005;86(Pt 9):2553–61. Epub 2005/08/16. 10.1099/vir.0.81021-0 . 16099914

[12] Chang JS, Chi SC. GHSC70 is involved in the cellular entry of nervous necrosis virus. J Virol. 2015;89(1):61–70. 10.1128/JVI.02523-14 25320288 PMC4301162

[13] Riblett AM, Blomen VA, Jae LT, Altamura LA, Doms RW, Brummelkamp TR, et al A haploid genetic screen identifies heparan sulfate proteoglycans supporting rift valley fever virus infection. J Virol. 2016;90(3):1414–23. Epub 2015/11/20. 10.1128/JVI.02055-15 26581979 PMC4719632

[14] Li J, Buchner J. Structure, function and regulation of the hsp90 machinery. Biomed J. 2013;36(3):106–17. Epub 2013/06/29. 10.4103/2319-4170.113230 . 23806880

[15] Momose F, Naito T, Yano K, Sugimoto S, Morikawa Y, Nagata K. Identification of Hsp90 as a stimulatory host factor involved in influenza virus RNA synthesis. J Biol Chem. 2002;277(47):45306–14. 10.1074/jbc.M206822200 PubMed PMID: WOS:000179404800092. 12226087

[16] Altieri DC, Stein GS, Lian JB, Languino LR. TRAP-1, the mitochondrial Hsp90. Bba-Mol Cell Res. 2012;1823(3):767–73. 10.1016/j.bbamcr.2011.08.007 PubMed PMID: WOS:000301628700019. 21878357 PMC3263322

[17] Marzec M, Eletto D, Argon Y. GRP94: An HSP90-like protein specialized for protein folding and quality control in the endoplasmic reticulum. Bba-Mol Cell Res. 2012;1823(3):774–87. 10.1016/j.bbamcr.2011.10.013 PubMed PMID: WOS:000301628700020. 22079671 PMC3443595

[18] Chen B, Piel WH, Gui L, Bruford E, Monteiro A. The HSP90 family of genes in the human genome: insights into their divergence and evolution. Genomics. 2005;86(6):627–37. Epub 2005/11/05. 10.1016/j.ygeno.2005.08.012 . 16269234

[19] Geller R, Taguwa S, Frydman J. Broad action of Hsp90 as a host chaperone required for viral replication. Biochim Biophys Acta. 2012;1823(3):698–706. 10.1016/j.bbamcr.2011.11.007 22154817 PMC3339566

[20] Wang Y, Li Y, Ding T. Heat shock protein 90beta in the Vero cell membrane binds Japanese encephalitis virus. Int J Mol Med. 2017;40(2):474–82. 10.3892/ijmm.2017.3041 28656253 PMC5505021

[21] Wang RY, Kuo RL, Ma WC, Huang HI, Yu JS, Yen SM, et al Heat shock protein-90-beta facilitates enterovirus 71 viral particles assembly. Virology. 2013;443(2):236–47. Epub 2013/05/29. 10.1016/j.virol.2013.05.001 . 23711381

[22] Wang Y, Wang R, Li F, Wang Y, Zhang Z, Wang Q, et al Heat-shock protein 90alpha is involved in maintaining the stability of VP16 and VP16-mediated transactivation of alpha genes from herpes simplex virus-1. Mol Med. 2018;24(1):65 Epub 2018/12/24. 10.1186/s10020-018-0066-x 30577726 PMC6303900

[23] Lin TW, Lo CW, Lai SY, Fan RJ, Lo CJ, Chou YM, et al Chicken heat shock protein 90 is a component of the putative cellular receptor complex of infectious bursal disease virus. J Virol. 2007;81(16):8730–41. 10.1128/JVI.00332-07 17522206 PMC1951386

[24] Lee SH, Song R, Lee MN, Kim CS, Lee H, Kong YY, et al A molecular chaperone glucose-regulated protein 94 blocks apoptosis induced by virus infection. Hepatology. 2008;47(3):854–66. 10.1002/hep.22107 PubMed PMID: WOS:000253698900012. 18273841

[25] Borrow P, Oldstone MB. Characterization of lymphocytic choriomeningitis virus-binding protein(s): a candidate cellular receptor for the virus. J Virol. 1992;66(12):7270–81. Epub 1992/12/01. 1331520 10.1128/jvi.66.12.7270-7281.1992 PMC240431

[26] Reyes-Del Valle J, Chavez-Salinas S, Medina F, Del Angel RM. Heat shock protein 90 and heat shock protein 70 are components of dengue virus receptor complex in human cells. J Virol. 2005;79(8):4557–67. 10.1128/JVI.79.8.4557-4567.2005 15795242 PMC1069525

[27] Carette JE, Raaben M, Wong AC, Herbert AS, Obernosterer G, Mulherkar N, et al Ebola virus entry requires the cholesterol transporter Niemann-Pick C1. Nature. 2011;477(7364):340–3. Epub 2011/08/26. 10.1038/nature10348 21866103 PMC3175325

[28] Iwamoto T, Okinaka Y, Mise K, Mori KI, Arimoto M, Okuno T, et al Identification of host-specificity determinants in betanodaviruses by using reassortants between striped jack nervous necrosis virus and sevenband grouper nervous necrosis virus. J Virol. 2004;78(3):1256–62. 10.1128/jvi.78.3.1256-1262.2004 PubMed PMID: WOS:000188205900020. 14722280 PMC321384

[29] Zhang M, Wu Q, Chen Y, Duan M, Tian G, Deng X, et al Inhibition of proanthocyanidin A2 on porcine reproductive and respiratory syndrome virus replication in vitro. PLoS One. 2018;13(2):e0193309 10.1371/journal.pone.0193309 . 29489892 PMC5831109

[30] Prajapati M, Alfred N, Dou YX, Yin XP, Prajapati R, Li YM, et al Host cellular receptors for the peste des petits ruminant virus. Viruses-Basel. 2019;11(8). doi: ARTN 729 10.3390/v11080729 PubMed PMID: WOS:000482993900044. 31398809 PMC6723671

[31] Gerna G, Kabanova A, Lilleri D. Human cytomegalovirus cell tropism and host cell receptors. Vaccines (Basel). 2019;7(3). Epub 2019/07/25. 10.3390/vaccines7030070 . 31336680 PMC6789482

[32] Haase M, Fitze G. HSP90AB1: Helping the good and the bad. Gene. 2016;575(2 Pt 1):171–86. Epub 2015/09/12. 10.1016/j.gene.2015.08.063 26358502 PMC5675009

[33] Hainzl O, Lapina MC, Buchner J, Richter K. The charged linker region is an important regulator of Hsp90 function. J Biol Chem. 2009;284(34):22559–67. Epub 2009/06/26. 10.1074/jbc.M109.031658 19553666 PMC2755663

[34] Chen NC, Yoshimura M, Guan HH, Wang TY, Misumi Y, Lin CC, et al Crystal structures of a piscine betanodavirus: Mechanisms of capsid assembly and viral infection. PLoS Pathog. 2015;11(10):e1005203 10.1371/journal.ppat.1005203 26491970 PMC4619592

[35] Tsou YL, Lin YW, Chang HW, Lin HY, Shao HY, Yu SL, et al Heat shock protein 90: role in enterovirus 71 entry and assembly and potential target for therapy. PLoS One. 2013;8(10):e77133 Epub 2013/10/08. 10.1371/journal.pone.0077133 24098578 PMC3788750

[36] Hung CY, Tsai MC, Wu YP, Wang RY. Identification of heat-shock protein 90 beta in Japanese encephalitis virus-induced secretion proteins. J Gen Virol. 2011;92(Pt 12):2803–9. Epub 2011/08/05. 10.1099/vir.0.033993-0 . 21813703

[37] Nishizawa T, Furuhashi M, Nagai T, Nakai T, Muroga K. Genomic classification of fish nodaviruses by molecular phylogenetic analysis of the coat protein gene. Appl Environ Microbiol. 1997;63(4):1633–6. Epub 1997/04/01. 9097459 10.1128/aem.63.4.1633-1636.1997 PMC168456

[38] Ito Y, Okinaka Y, Mori KI, Sugaya T, Nishioka T, Oka M, et al Variable region of betanodavirus RNA2 is sufficient to determine host specificity. Dis Aquat Organ. 2008;79(3):199–205. 10.3354/dao01906 PubMed PMID: WOS:000256419900004. 18589996

[39] Adachi K, Ichinose T, Watanabe K, Kitazato K, Kobayashi N. Potential for the replication of the betanodavirus redspotted grouper nervous necrosis virus in human cell lines. Arch Virol. 2008;153(1):15–24. Epub 2007/10/02. 10.1007/s00705-007-1051-9 . 17906832 PMC7086817

[40] Nagai T, Nishizawa T. Sequence of the non-structural protein gene encoded by RNA1 of striped jack nervous necrosis virus. J Gen Virol. 1999;80 (Pt 11):3019–22. 10.1099/0022-1317-80-11-3019 . 10580064

[41] Huang RQ, Zhu GH, Zhang J, Lai YX, Xu Y, He JG, et al Betanodavirus-like particles enter host cells via clathrin-mediated endocytosis in a cholesterol-, pH- and cytoskeleton-dependent manner. Vet Res. 2017;48. doi: ARTN 8 10.1186/s13567-017-0454-1 PubMed PMID: WOS:000394187200001. 28179028 PMC5299686

[42] Eisenberg E, Greene LE. Multiple roles of auxilin and Hsc70 in clathrin-mediated endocytosis. Traffic. 2007;8(6):640–6. 10.1111/j.1600-0854.2007.00568.x PubMed PMID: WOS:000246626300002. 17488288

[43] Cho HJ, Kim GH, Park SH, Hyun JY, Kim NK, Shin I. Probing the effect of an inhibitor of an ATPase domain of Hsc70 on clathrin-mediated endocytosis. Mol Biosyst. 2015;11(10):2763–9. 10.1039/c4mb00695j PubMed PMID: WOS:000361547500015. 25728281

[44] Fitter S, Gronthos S, Ooi SS, Zannettino AC. The mesenchymal precursor cell marker antibody STRO-1 binds to cell surface heat shock cognate 70. Stem Cells. 2017;35(4):940–51. Epub 2016/12/28. 10.1002/stem.2560 . 28026090

[45] Sidera K, Samiotaki M, Yfanti E, Panayotou G, Patsavoudi E. Involvement of cell surface HSP90 in cell migration reveals a novel role in the developing nervous system. J Biol Chem. 2004;279(44):45379–88. Epub 2004/08/11. 10.1074/jbc.M405486200 . 15302889

[46] Snigireva AV, Vrublevskaya VV, Skarga YY, Morenkov OS. The role of membrane-bound heat shock proteins Hsp90 in migration of tumor cells in vitro and involvement of cell surface heparan sulfate proteoglycans in protein binding to plasma membrane. Biofizika. 2016;61(2):328–36. Epub 2016/05/20. . 27192836

[47] Guerrero CA, Bouyssounade D, Zarate S, Isa P, Lopez T, Espinosa R, et al Heat shock cognate protein 70 is involved in rotavirus cell entry. J Virol. 2002;76(8):4096–102. Epub 2002/03/22. 10.1128/jvi.76.8.4096-4102.2002 11907249 PMC136078

[48] Joshi P, Stoddart CA. Impaired infectivity of ritonavir-resistant HIV is rescued by heat shock protein 90AB1. J Biol Chem. 2011;286(28):24581–92. 10.1074/jbc.M111.248021 PubMed PMID: WOS:000292547900008. 21602280 PMC3137033

[49] Joshi P, Sloan B, Torbett BE, Stoddart CA. Heat shock protein 90AB1 and hyperthermia rescue infectivity of HIV with defective cores. Virology. 2013;436(1):162–72. 10.1016/j.virol.2012.11.005 PubMed PMID: WOS:000314003800019. 23200770 PMC3563299

[50] Kawashima D, Kanda T, Murata T, Saito S, Sugimoto A, Narita Y, et al Nuclear transport of Epstein-Barr virus DNA polymerase is dependent on the BMRF1 polymerase processivity factor and molecular chaperone Hsp90. J Virol. 2013;87(11):6482–91. Epub 2013/04/05. 10.1128/JVI.03428-12 23552409 PMC3648106

[51] Yi M, Hong N, Hong Y. Generation of medaka fish haploid embryonic stem cells. Science. 2009;326(5951):430–3. 10.1126/science.1175151 . 19833967

[52] Le Y, Li Y, Jin Y, Jia P, Jia K, Yi M. Establishment and characterization of a brain cell line from sea perch, *Lateolabrax japonicus*. In Vitro Cell Dev Biol Anim. 2017;53(9):834–40. 10.1007/s11626-017-0185-7 . 28707225

[53] Jia P, Jia KT, Yi MS. Complete genome sequence of a fish nervous necrosis virus isolated from sea perch (Lateolabrax japonicus) in China. Microbiol Resour Ann. 2015;3(3). doi: ARTN e00048-15 10.1128/genomeA.00048-15. PubMed PMID: WOS:000460631200001. PMC4457048 26044411

[54] Jia KT, Wu YY, Liu ZY, Mi S, Zheng YW, He J, et al Mandarin fish caveolin 1 interaction with major capsid protein of infectious spleen and kidney necrosis virus and its role in early stages of infection. J Virol. 2013;87(6):3027–38. 10.1128/JVI.00552-12 PubMed PMID: WOS:000315348500005. 23283951 PMC3592132

[55] Pidasheva S, Canaff L, Simonds WF, Marx SJ, Hendy GN. Impaired cotranslational processing of the calcium-sensing receptor due to signal peptide missense mutations in familial hypocalciuric hypercalcemia. Hum Mol Genet. 2005;14(12):1679–90. Epub 2005/05/10. 10.1093/hmg/ddi176 . 15879434

[56] Livak KJ, Schmittgen TD. Analysis of relative gene expression data using real-time quantitative PCR and the 2(T)(-Delta Delta C) method. Methods. 2001;25(4):402–8. 10.1006/meth.2001.1262 PubMed PMID: WOS:000173949500003. 11846609

[57] Dong S, Kang M, Wu X, Ye T. Development of a promising fish model (Oryzias melastigma) for assessing multiple responses to stresses in the marine environment. Biomed Res Int. 2014;2014:563131 Epub 2014/04/12. 10.1155/2014/563131 24724087 PMC3958766

