# Supplementary figures and images for: Marine medaka heat shock protein 90ab1 is a receptor for red-spotted grouper nervous necrosis virus and promotes virus internalization through clathrin-mediated endocytosis
Source: PLoS Pathog. 2020 Jul 8;16(7):e1008668. doi: 10.1371/journal.ppat.1008668 (PMC7371229; doi:10.1371/journal.ppat.1008668)

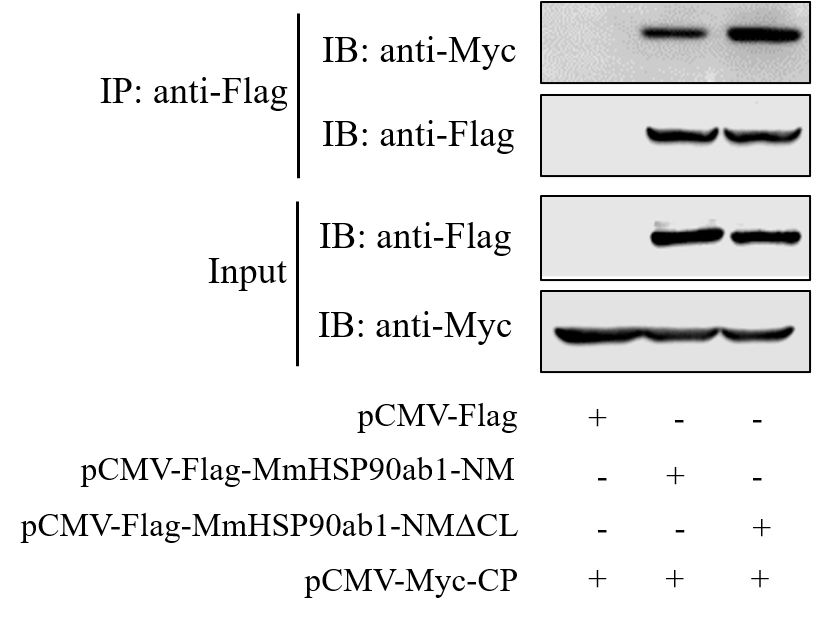

Supplement: S1 Fig — HEK293T cells were cotransfected with pCMV-Myc-CP and pCMV-Flag-MmHSP90ab1-NM or pCMV-Flag-MmHSP90ab1-NMΔ CL for 48 h, respectively. Cell lysates were immunoprecipitated with anti-Flag abs. The immunoprecipitates and input were immunoblotted with the indicated abs. (TIF) [file ppat.1008668.s003.tif]
